# Effect of Chromium on Corrosion Behavior of P110 Steels in CO_2_-H_2_S Environment with High Pressure and High Temperature

**DOI:** 10.3390/ma9030200

**Published:** 2016-03-16

**Authors:** Jianbo Sun, Chong Sun, Xueqiang Lin, Xiangkun Cheng, Huifeng Liu

**Affiliations:** School of Mechanical and Electronic Engineering, China University of Petroleum, Qingdao 266580, China; sunchongupc@126.com (C.S.); linxueqiang@126.com (X.L.); xiangkun_upc@163.com (X.C.); liuhuifeng1203@163.com (H.L.)

**Keywords:** low-chromium steel, corrosion scale, weight loss, scanning electron microscope, X-ray photoelectron spectroscopy, CO_2_/H_2_S corrosion

## Abstract

The novel Cr-containing low alloy steels have exhibited good corrosion resistance in CO_2_ environment, mainly owing to the formation of Cr-enriched corrosion film. In order to evaluate whether it is applicable to the CO_2_ and H_2_S coexistence conditions, the corrosion behavior of low-chromium steels in CO_2_-H_2_S environment with high pressure and high temperature was investigated using weight loss measurement and surface characterization. The results showed that P110 steel suffered localized corrosion and both 3Cr-P110 and 5Cr-P110 steels exhibited general corrosion. However, the corrosion rate of 5Cr-P110 was the highest among them. The corrosion process of the steels was simultaneously governed by CO_2_ and H_2_S. The outer scales on the three steels mainly consisted of FeS_1−*x*_ crystals, whereas the inner scales on Cr-containing steels comprised of amorphous FeS_1−*x*_, Cr(OH)_3_ and FeCO_3_, in contrast with the amorphous FeS_1−*x*_ and FeCO_3_ mixture film of P110 steel. The more chromium the steel contains, the more chromium compounds the corrosion products contain. The addition of chromium in steels increases the uniformity of the Cr-enriched corrosion scales, eliminates the localized corrosion, but cannot decrease the general corrosion rates. The formation of FeS_1−*x*_ may interfere with Cr-enriched corrosion scales and lowering the corrosion performance of 3Cr-P110 and 5Cr-P110 steels.

## 1. Introduction

The CO_2_/H_2_S corrosion problems of oil country tubular goods (OCTG) become increasingly prominent with the exploitation of oil and gas field under high temperature and high CO_2_ and/or H_2_S pressure [1,2,3,4,5,6]. Corrosion resistant alloys (CRAs), such as stainless steels and high-nickel alloys, have been developed to mitigate the CO_2_/H_2_S corrosion long time ago, but the high cost constrains their application in oil and gas field containing CO_2_ and/or H_2_S. Therefore, carbon and low alloy steel are still cost-effective materials used for tubings and pipelines despite of the shortcoming of high corrosion rate, and great efforts have been made to increase its corrosion resistance [4,5,6,7,8,9]. In recent years, the novel Cr-containing low alloy steels have been developed to balance the cost advantage and corrosion resistance between carbon steel and CRAs [5,6,7,8,9,10,11,12,13]. Many studies indicate that the low Cr alloy steels with 3–5 wt % Cr can not only remarkably reduce the CO_2_ corrosion rate, but also avoid localized corrosion in CO_2_ environment, mainly due to the formation of the amorphous Cr(OH)_3_ in the scales on low Cr alloy steel [8,9,10,11,12,13]. Sun *et al.* [12] reported that, with an addition of 3% chromium in X65 steel, the corrosion rate dropped significantly from 11.59 to 1.57 mm/y and the localized corrosion was eliminated due to the formation of the FeCO_3_ and Cr(OH)_3_ scale mixtures on low-chromium steels under 1 MPa CO_2_. Kermani *et al.* [8] and Pigliacampo *et al.* [14] also found the Cr-enriched protective layer on 3 wt % Cr and 5 wt % Cr steels in sweet downhole production conditions.

In view of the good corrosion resistance exhibited by the low Cr alloy steel in a CO_2_ environment, researchers began to explore whether it is applicable to the CO_2_ and H_2_S coexistence conditions. Kermani *et al.* [8] reported the satisfactory sulfide stress cracking (SSC) performance of 3 wt % Cr tubing and improved corrosion rates of some 2.5–6 times than that of L80 in CO_2_ (0.095 MPa) environment containing trace of H_2_S (0.005 MPa). Many literatures have indicated that low levels of H_2_S functions well in reducing CO_2_ corrosion because the sulfide scales can give protection to the underlying steel. However, with the introduction of trace of H_2_S (sour systems), it is supposed that the formation of FeS may interfere with Cr-enriched FeCO_3_, hence lowering the corrosion performance of 3 wt % Cr steel. Therefore, in terms of corrosion performance, the 3 wt % Cr steel exhibits greater superiority relative to carbon and low alloy steels in sweet system than in sour system [14].

The competitive formation of iron sulfide and iron carbonate on carbon and low alloy steel is one of the important factors to affect the corrosion rate [15,16,17]. It is acknowledged that iron carbonate (FeCO_3_) is a typical CO_2_ corrosion product, and, due to the influence of the factors such as temperature, pH and H_2_S concentration, the types of iron sulfide products formed by H_2_S corrosion are more complex, including mackinawite, pyrrhotite, troilite, cubic ferrous sulfide, pyrite, smythite, and greigite *etc.* [2,18,19,20]. Many studies have illustrated that the presence of H_2_S in a CO_2_ environment could either accelerate or mitigate the corrosion of carbon steel, depending on the H_2_S partial pressure and the environmental conditions [21,22,23]. The CO_2_/H_2_S corrosion mechanism of carbon steel can be identified by the CO_2_/H_2_S pressure ratio (*P*_CO_2__/*P*_H_2_S_) [24,25,26]. For example, Pots *et al.* [25] reported that when *P*_CO_2__/*P*_H_2_S_ < 20, H_2_S controlled the corrosion process; when *P*_CO_2__/*P*_H_2_S_ was between 20 and 500, the corrosion process was simultaneously controlled by CO_2_ and H_2_S, and CO_2_ had a dominant control on the corrosion when *P*_CO_2__/*P*_H_2_S_ > 500. Srimivasan *et al.* [26] showed that when *P*_CO_2__/*P*_H_2_S_ > 200, CO_2_ played a primary role in this system. Mackinawite could form on the steel surface at the temperature below 120 °C to mitigate corrosion. However, when *P*_CO_2__/*P*_H_2_S_ < 200, the iron sulfide tended to deposit prior to iron carbonate, and the corrosion of carbon steel was determined by the stability and the protective performance of iron sulfide and iron carbonate.

It must be emphasized that mild steel tends to suffer from localized attack, such as ringworm corrosion and mesa corrosion, which cannot be controlled by inhibitors in the environment with a H_2_S partial pressure higher than 0.02 MPa [10]. In the presence of a high concentration of H_2_S, the corrosion rate may be higher than predicted by means of CO_2_ corrosion prediction models [1]. H_2_S may form non-protective layers and catalyze the anodic dissolution of bare steel [1].

As for the novel low Cr steel, the interaction between H_2_S/CO_2_ and steel is more complex. At present, there is still very limited study on the CO_2_/H_2_S corrosion behavior and mechanism of low Cr alloy steel. Therefore, it is difficult to determine whether the low Cr alloy steel could be applied to CO_2_ and H_2_S coexistence environment, especially under the high partial pressure of CO_2_ and H_2_S. Against this background, the aim of this work is to investigate the corrosion behavior and analyze the characteristics of the corrosion scale of low Cr alloy steel in a CO_2_-H_2_S environment with high pressure and high temperature.

## 2. Experimental Procedure

### 2.1. Material and Pretreatment

The P110, 3Cr-P110, and 5Cr-P110 tube steels, with chemical compositions shown in Table 1, were used in this study. The specimens were machined into a size of 35 mm × 15 mm × 3 mm. Before the tests, the working surface of each specimen was abraded with silicon carbide paper of decreasing roughness (up to 800 grit), rinsed with deionized water, degreased with acetone and dried in air. The four parallel specimens for each test were weighed using an electronic balance with a precision of 0.1 mg, installed in a modified rotating polytetrafluoroethylene (PTFE) holder and then stored in a desiccator.

The immersion corrosion test solution, a 3.5 wt % NaCl solution, was prepared with analytical grade reagents and deionized water.

### 2.2. Weight Loss Test

To investigate the corrosion rate and corrosion morphology of tube steels in CO_2_-H_2_S environment, immersion corrosion tests were carried out in a 3 L autoclave featuring high temperature and high pressure. Prior to the tests, the solution was purged with highly-purified N_2_ to deoxidize for 12 h. The specimens were immersed into solution as soon as the solution was added into the autoclave, and then purging N_2_ was used to remove the air for 2 h immediately after the autoclave was closed. After that, the vent valve was closed. The solution was heated to 90 °C, and then the CO_2_/H_2_S mixture gases (*P*_CO_2__/*P*_H_2_S_ = 25) were injected to autoclave to reach a total pressure of 5.2 MPa (*P*_CO_2__ = 5 MPa, *P*_H_2_S_ = 0.2 MPa). The flow rate was 1 m/s at specimen surface. The tests were carried out for 360 h.

After corrosion tests, the specimens were taken out of the autoclave, rinsed in deionized water, dehydrated in alcohol and dried in air, respectively. One of the four specimens was retained for surface characterization of corrosion scales. The rest three specimens were descaled in the solution consisting of hydrochloric acid (100 mL, density is 1.19 g/mL), hexamethylene tetramine (5 g), and deionized water (900 mL) at room temperature, and then processed as above. After that, the specimens were weighed again to determine the weight loss. The corrosion rate was calculated through the following equation:

(1) $$V_{CR}=\frac{8.76\times 10^{4}\Delta W}{S\rho t}$$

where *V_CR_* is the corrosion rate, mm/y; *W* is the weight loss, g; *S* is the exposed surface area of specimen, cm^2^; *ρ* is the density of specimen, g/cm^3^; *t* is the corrosion time, h; 8.76 × 10^4^ is the unit conversion constant. The average corrosion rate with error bars was calculated from the three parallel specimens for each test.

### 2.3. Characterization of the Corrosion Scale

The surface and cross-sectional morphologies of the corrosion scales were observed using scanning electron microscope (SEM). The elemental compositions of the corrosion scales were analyzed using energy dispersive spectroscopy (EDS) with an acceleration voltage of 15 kV. The phase compositions of the corrosion scales were identified by means of X-ray diffraction (XRD) with a Cu Kα X-ray source operated at 40 kV and 150 mA, and the surface chemistry of the corrosion scales were also measured by X-ray photoelectron spectroscopy (XPS) with an Al Kα (*hv* = 1486.6 eV) X-ray source.

## 3. Results

### 3.1. Corrosion Rate and Corrosion Form

**Figure 1:**
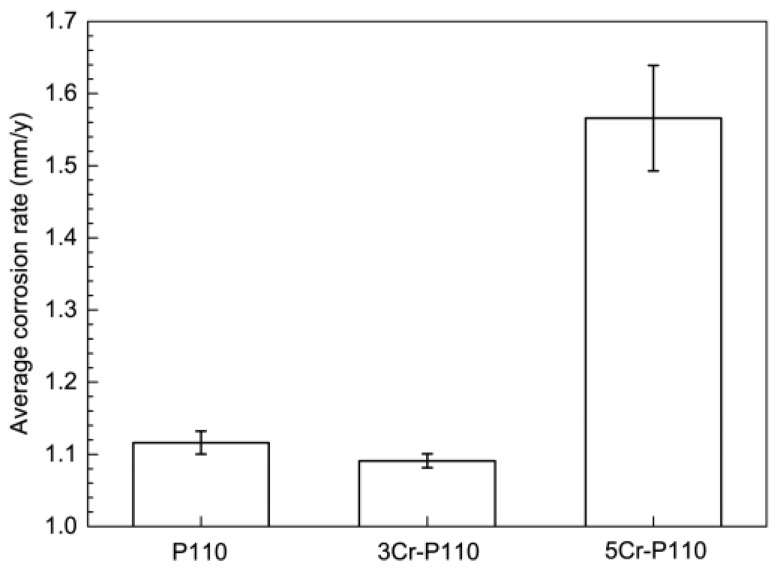
Average corrosion rates of P110, 3Cr-P110 and 5Cr-P110 tube steels in 3.5 wt % NaCl solution with CO_2_ and H_2_S (*P*_CO_2__ = 5 MPa, *P*_H_2_S_ = 0.2 MPa, 90 °C, 1 m/s, 360 h). The error bar of average corrosion rate was calculated from the three parallel specimens for each test.

Figure 1 presents the average corrosion rates of tested tube steels after immersion corrosion tests in a CO_2_-H_2_S environment. It can be seen that the corrosion rate of 5Cr-P110 (1.57 mm/y) was the highest among the three steels, and the corrosion rate of 3Cr-P110 (1.08 mm/y) was approximate to that of P110 (1.12 mm/y), taking into account the experimental error. This suggests that the corrosion resistance of P110 tube steel cannot be significantly improved by increasing Cr content in a CO_2_-H_2_S condition (*P*_CO_2__ = 5 MPa, *P*_H_2_S_ = 0.2 MPa). Therefore, although the low-chromium steel has exhibited eminent corrosion resistance in CO_2_ environment, especially when Cr content is 3–5 wt % in the steel [11,12], it may not be necessarily applicable to the CO_2_-H_2_S condition with high pressure. That is to say, for low Cr steel, the corrosion performance varies depending on different operational conditions. In a CO_2_ environment, the protection is provided through the formation of a Cr-enriched FeCO_3_ corrosion product (Cr oxi-hydroxide) [9,10,11,12,13]. The more chromium the steel contains, the more chromium compounds the corrosion products contain, and the better the protection is. However, under test condition, H_2_S might play a significant role in determining the type and properties of the corrosion scales, *i.e.*, gradually undermining the corrosion scales and reducing their protective performance [1,14].

**Figure 2:**
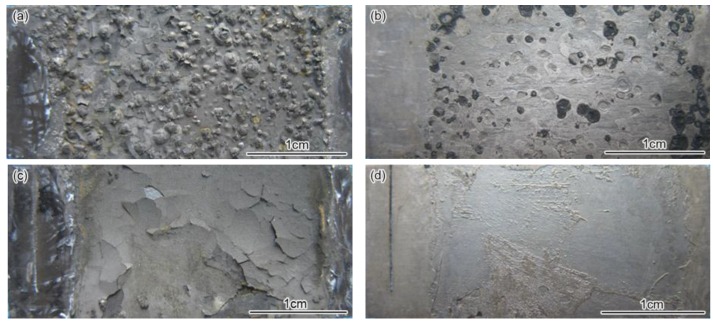

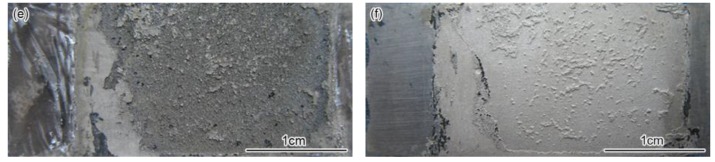
Macroscopic surface morphology of the tube steels before (**a**,**c**,**e**) and after (**b**,**d**,**f**) the removal of corrosion scales (*P*_CO_2__ = 5 MPa, *P*_H_2_S_ = 0.2 MPa, 90 °C, 1 m/s, 360 h, 3.5 wt % NaCl): (a,b) P110; (c,d) 3Cr-P110 and (e,f) 5Cr-P110.

Figure 2 shows the macroscopic surface morphology of the three steels before and after the removal of corrosion scales. As exhibited in the figures, the P110 steel surface were covered with tumor-like corrosion products (Figure 2a), and after removal of the corrosion scales, the positions where the “tuberculation” covered suffered shallow mesa attack (Figure 2b). This indicates that there is probably some connection between localized corrosion and the corrosion scale at that location. In contrast, the corrosion scales were relatively flat on 3Cr-P110 and 5Cr-P110 steels surface with a non-adhesive outer layer (Figure 2c,e). After the specimens were taken out from solution, the outer layer cracked due to dehydration and partly peeled-off the 3Cr-P110 steel surface (Figure 2c), and most of the outer layer scales peeled off the 5Cr-P110 steel surface (Figure 2e). 3Cr-P110 and 5Cr-P110 steels were subject to general corrosion (Figure 2d,f), indicating that the addition of 3–5 wt % Cr into carbon steel improves the localized corrosion resistance in CO_2_-H_2_S environment, which is consistent with the case in CO_2_ environment [11,12,13]. However, the corrosion rates of Cr-containing steels did not decrease, suggesting that the addition of chromium could increase the uniformity of the corrosion scale but not improve the diffusion resistance to corrosive ions.

### 3.2. The Composition and Elements Distribution of Corrosion Film

**Figure 3:**
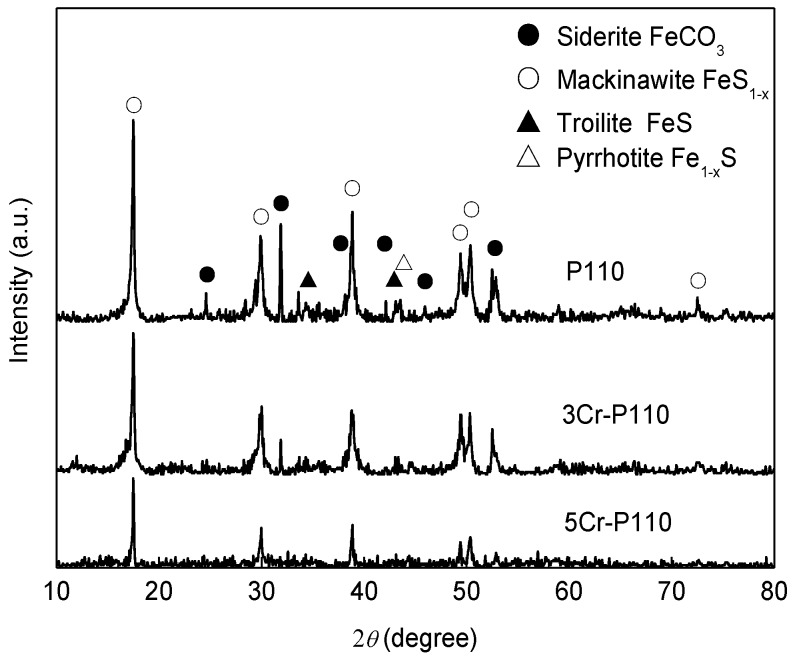
X-ray diffraction (XRD) spectra of corrosion scales on the steels with different Cr contents.

Figure 3 shows the XRD spectra of the corrosion scales on the steels. It can be seen that the crystals in the scales of P110 steel mainly comprised FeS_1−*x*_ and FeCO_3_, with a small amount of FeS and Fe_1−*x*_ S. However the main crystals in the scales of 3Cr-P110 and 5Cr-P110 steels were FeS_1−*x*_ with only a small amount of FeCO_3_ crystals detected. The content of FeCO_3_ crystals in corrosion products gradually reduced in the order of P110, 3Cr-P110 and 5Cr-P110. The crystalline state of FeCO_3_ is predominantly determined by the pH [9]. High pH value causes the formation of FeCO_3_ crystal while low pH results in amorphous FeCO_3_. Therefore, the reduction of FeCO_3_ crystals is probably, to some extent, related to the pH of the solution in the proximity of the solution/scale or solution/metal interface.

**Figure 4:**
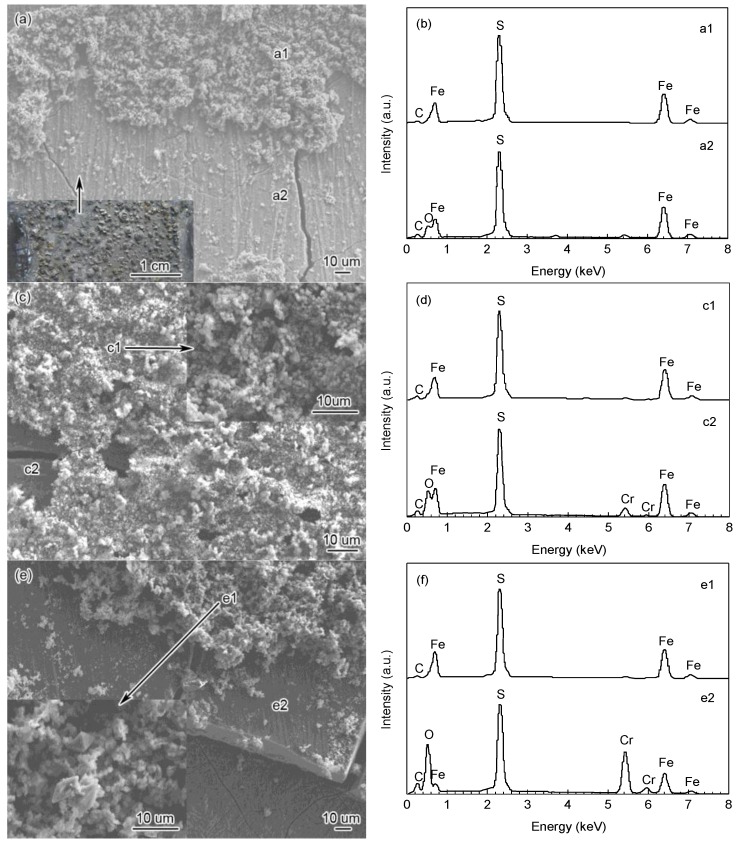
(**a**,**c**,**e**) Scanning electron microscope (SEM) images and (**b**,**d**,**f**) energy dispersive spectroscopy (EDS) analysis of the surface products on (a,b) P110; (c,d) 3Cr-P110 and (e,f) 5Cr-P110 tube steels in 3.5 wt % NaCl solution with CO_2_ and H_2_S: (b) denoted by a1 and a2 in (a); (d) denoted by c1 and c2 in (c) and (f) denoted by e1 and e2 in (e) (*P*_CO_2__ = 5 MPa, *P*_H_2_S_ = 0.2 MPa, 90 °C, 1 m/s, 360 h).

SEM surface morphology of corrosion scales on the steels are shown in Figure 4. As exhibited in Figure 4a,c,e, the corrosion scales on the three steels showed similar morphology. The outer corrosion layers comprised tiny crystalline products, and the result of EDS analysis indicated that the outer layer scales mainly consisted of Fe and S elements (Figure 4b,d,f). It was concluded that these tiny crystalline products are mainly FeS_1−*x*_. However, the inner scales showed amorphous characteristic with cracks caused by surface dehydration, and all contained Fe, S and O elements. In addition, the inner scales of 3Cr-P110 and 5Cr-P110 steels contained abundant amounts of Cr (19.99 wt % and 28.55 wt %, respectively). Therefore, there must be some compounds, such as Cr-compounds, which were undetected by XRD.

**Figure 5:**
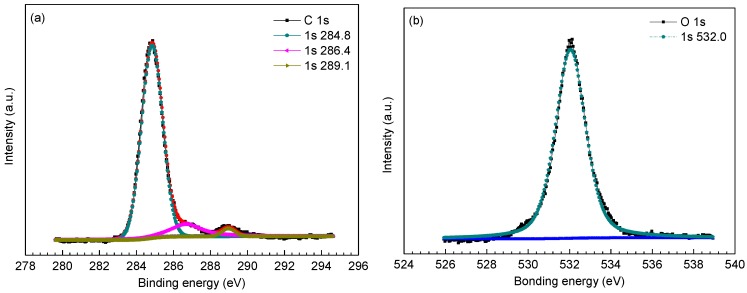

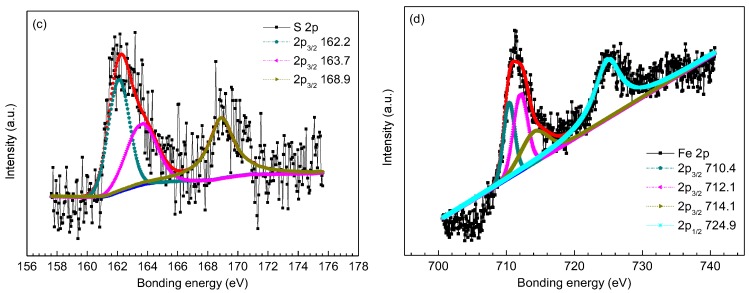
X-ray photoelectron spectroscopy (XPS) spectra and decomposition of peaks for different elements of the inner scale on P110 steel: (**a**) C 1s; (**b**) O 1s; (**c**) S 2p; and (**d**) Fe 2p.

**Figure 6:**
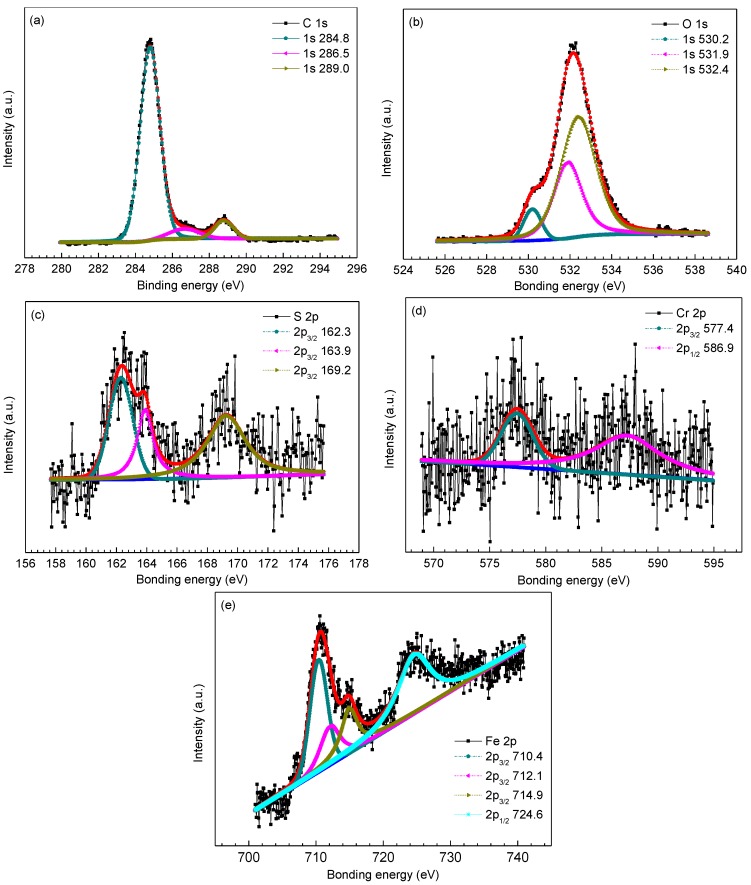
X-ray photoelectron spectroscopy (XPS) spectra and decomposition of peaks for different elements of the inner scale on 3Cr-P110 steel: (**a**) C 1s; (**b**) O 1s; (**c**) S 2p; (**d**) Cr 2p; and (**e**) Fe 2p.

**Figure 7:**
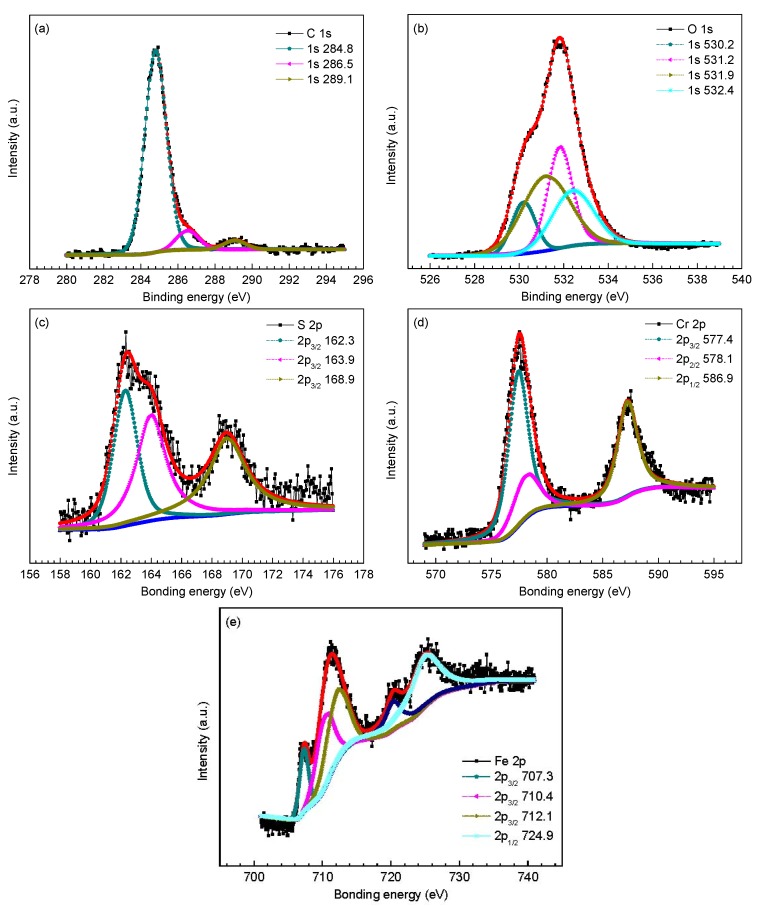
X-ray photoelectron spectroscopy (XPS) spectra and decomposition of peaks for different elements of the inner scale on 5Cr-P110 steel: (**a**) C 1s; (**b**) O 1s; (**c**) S 2p; (**d**) Cr 2p; and (**e**) Fe 2p.

To further determine the phase composition of inner scales, XPS analysis was employed to analyze the surface chemistry of amorphous compounds in the inner scales. Figure 5, Figure 6 and Figure 7 provides the high resolution XPS spectra of inner scales on P110, 3Cr-P110 and 5Cr-P110 steels. The elements of interest were Fe, O, S and C and, in the meantime, Cr was also investigated for 3Cr-P110 and 5Cr-P110 steels. Surface charging effects were compensated by referencing the binding energy to the C 1s line of the residual carbon set at 284.6 eV. The Gaussian–Lorentzian curves were used to fit the peaks. Binding energies of Fe 2p, O 1s, S 2p, and Cr 2p for inner scales on P110, 3Cr-P110, and 5Cr-P110 samples are summarized in Table 2.

Referring to Figure 5 and Table 2 [2,27,28,29], the results suggested that the inner scale of P110 steel mainly consisted of FeS_1−*x*_ and FeCO_3_. The S 2p_3/2_ peak at 163.7 eV corresponded to elemental sulfur which was detected because iron sulfide got oxidized while in air [3]. In addition, considering the limitation of the corrosion environment, the S 2p_3/2_ peak at a binding energy of 168.9 eV could be attributable to the adventitious SO_4_^2−^ [30].

The high resolution XPS spectra of inner scales on 3Cr-P110 and 5Cr-P110 steels are shown in Figure 6 and Figure 7, respectively. As exhibited in Figure 6 and Figure 7 and Table 2, the C 1s, S 2p, and Fe 2p scans for the inner scales on 3Cr-P110 and 5Cr-P110 steels reflected a similar composition of corrosion products compared to that of P110 steel, confirming the presence of FeS_1-x_ and FeCO_3_. A small difference was the Fe 2p_3/2_ peak at 707.3 eV for 5Cr-P110 steel associated with iron sulfide [31], as shown in Figure 7e. However, a noticeable difference existed in the O spectra for 3Cr-P110 and 5Cr-P110 steels compared to that of P110 steel, along with the existence of Cr_2_O_3_ and Cr(OH)_3_ [9,32], except for FeCO_3_ [27]. The Cr 2p peaks both revealed the existence of Cr(OH)_3_ [9,32,33] in 3Cr-P110 and 5Cr-P110 scales. The Cr_2_O_3_ [32] in 5Cr-P110 scale was believed to be the product of the dehydration of Cr(OH)_3_ upon removal from the system [13]. The compositions of the inner scale on 3Cr-P110 and 5Cr-P110 steels were similar, both primarily consisting of FeS_1−*x*_, FeCO_3_ and amorphous Cr(OH)_3_.

**Figure 8:**
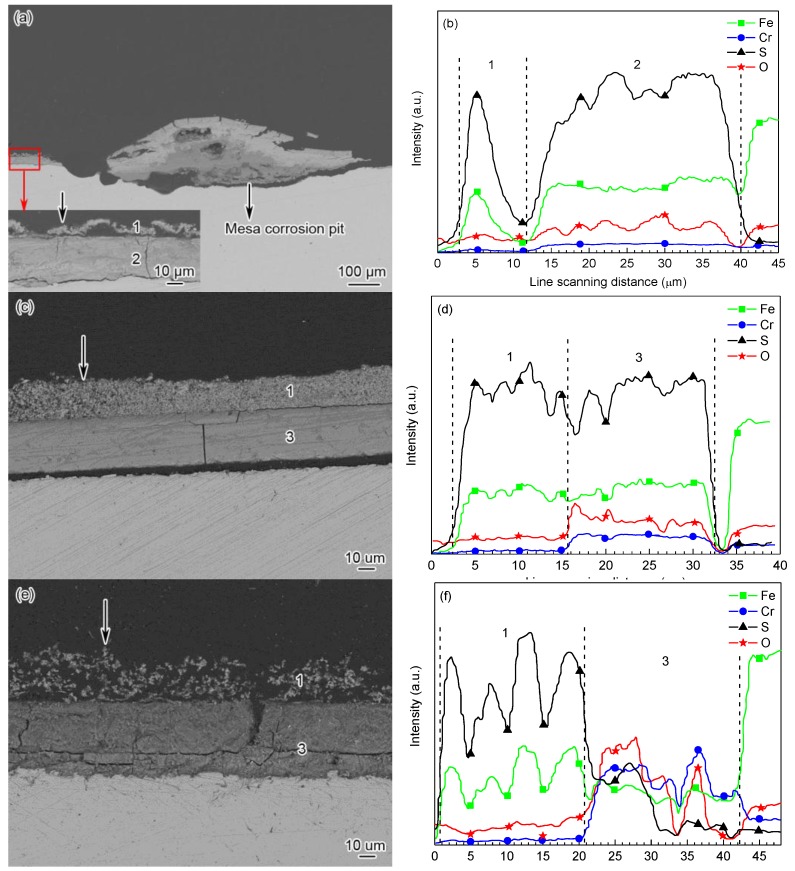
(**a**,**c**,**e**) cross-sectional backscattered electron images and (**b**,**d**,**f**) elemental distributions in cross-sections of the corrosion scales on (a,b) P110; (c,d) 3Cr-P110, and (e,f) 5Cr-P110 tube steels: (b) denoted by arrow in (a); (d) denoted by arrow in (c) and (f) denoted by arrow in (e) (1—FeS_1−*x*_; 2—FeS_1−*x*_ + FeCO_3_ and 3—FeS_1−*x*_ + Cr(OH)_3_ + FeCO_3_).

Figure 8 shows the cross-sectional backscattered electron images and EDS line scanning analysis of corrosion scales on the steels. As exhibited in Figure 8a,c,e, the scales on P110 (the position where P110 steel presented general corrosion morphology), 3Cr-P110 and 5Cr-P110 steels all had a two-layer structure after 360 h corrosion tests. It can be seen that the outer scales on three steels (Figure 8a–f) mainly contained Fe and S elements (FeS_1−*x*_), the inner scale on P110 steel (Figure 8a,b) mainly contained Fe, S, and O elements (FeS_1−*x*_ and FeCO_3_) and the inner scale on 3Cr-P110 (Figure 8c,d) and 5Cr-P110 (Figure 8e,f) steels mainly contained Fe, Cr, S and O elements (FeS_1−*x*_, Cr(OH)_3_, and FeCO_3_). The results were highly consistent with XRD and XPS analysis. As exhibited in Figure 8a, the outer FeS_1−*x*_ scale of P110 steel was very thin, uneven, and not closely attached to the inner scale with many large pores between inner and outer scales compared with that of 3Cr-P110 steel (Figure 8c). Whereas, for 5Cr-P110 steel, the outer FeS_1−*x*_ scale was porous and loose (Figure 8e) compared with that of 3Cr-P110 steel. This is the reason why most of the outer scale of 5Cr-P110 steel peeled off (Figure 2e).

According to the characteristics of corrosion scales on the tested steels, it is reasonable to believe that the corrosion process of the steels is simultaneously governed by CO_2_ and H_2_S under the test conditions (*P*_CO_2__/*P*_H_2_S_ = 25, 90 °C). It is well established that corrosion scales containing the same components could be extremely protective, very little so, or even corrosive depending on the location of these components [9,12,34,35,36,37]. The uneven formation and local damage of CO_2_ corrosion product film is the main reason for localized corrosion [38]. The mesa corrosion was observed from Figure 2b and Figure 8a, and the outer FeS_1−*x*_ scale disappeared in the regions where localized corrosion occurred. The local damage of corrosion scales on P110 steel provides the pathway for mass transfer of corrosive ions such as Cl^−^, HCO_3_^−^, HS^−^, and H^+^, which are sufficient to cause the onset of internal acidification [34], thus accelerating the dissolution of metal at the location. As the corrosion proceeds, a mass of corrosion products is formed in corrosion pits, thus forming the tumor-like corrosion products on the steel (Figure 2a and Figure 8a). However, for the Cr-containing steel, the formation of uniform corrosion scales inhibits the localized corrosion, which may be related to the high Cr content in corrosion films.

### 3.3. The Effect of Cr Content on Formation of Corrosion Scale and Its Relation to Corrosion

It is well established that driving force for precipitation is the supersaturation of corrosion products in CO_2_-H_2_S environment, which depends on both the carbon steel characteristics (microstructure, heat treatment history, alloying elements) and environmental variables (solution pH, temperature, solution composition, flow rate, *etc.*) [1,35]. As mentioned earlier, FeCO_3_, Fe*_y_*S*_x_* and Cr(OH)_3_ were all detected in the corrosion scales. The competitive deposition of FeCO_3_, Fe*_y_*S*_x_*, and Cr(OH)_3_ results in the mixed films which play an important role in determining the corrosion form and corrosion rate. 

In this study, H_2_S can increase CO_2_ corrosion by promoting anodic dissolution through sulfide adsorption and lowering pH and chromium can provide additional anodic reaction for Cr-containing steels. Therefore, both P110 steel and Cr-containing steels can be corroded rapidly at the early stage of the immersion period. Then, the supersaturation is so high that a high nucleation rate may emerge, causing an amorphous corrosion film formation on the steel surface. It can be seen from the cross-sections (Figure 8) that all the inner layers attached directly to the steel surface were apparently denser than outer layers, indicating a better protective performance of inner layers than outer layers. The inner scales acted as a diffusion barrier restricting the diffusion of reactive species and lowering the corrosion rates with time. It can also be seen that the inner layer on 5Cr-P110 was obviously not as compact as those on P110 and 3Cr-P110, which goes against the results in sweet system [12]. It suggested [14] that Fe*_y_*S*_x_* interfered with FeCO_3_ and Cr(OH)_3_ mixed film and reduced its protective properties especially for 5Cr-P110. Therefore, 5Cr-P110 had the highest corrosion rate, which may be attributable to the excessive chromium in 5Cr-P110 steel. According to the EDS line scanning analysis along the vertical direction of the cross-section (Figure 8b,d,f), the Cr content increased and S content decreased obviously in the inner scale on 5Cr-P110 steel compared with those in the inner scales on P110 and 3Cr-P110 steels. Since Cr(OH)_3_ is more stable than FeCO_3_ and Fe*_y_*S*_x_*, thermodynamically, the enrichment of Cr in the amorphous inner layers is due to the dissolution of FeCO_3_ [13,39] and Fe*_y_*S*_x_* in the scale, but it is unclear how H_2_S interferes with the Cr-enriched corrosion film formation and, hence, relatively lowers the protection properties of the films.

At the later stage of the immersion period, the outer FeS_1−*x*_ layer with a small crystalline form precipitated on the inner layers. However, the outer FeS_1−*x*_ scales of the three steels are strikingly different, such as the thickness and compactness, probably because of the the amorphous Cr(OH)_3_ in the inner scales. Guo *et al.* [9] found that the Cr content in steel had a significant impact on the *in situ* pH value of solution in the proximity of the scale/solution interface. The pH value can be reduced due to the formation of Cr(OH)_3_ by Equation (2) [9] or to the hydrolysis of Cr^3+^ ions by Equation (3) [40]. The higher the Cr content is, the lower the pH value can be achieved at the solution/scale interface. Therefore, the pH value in the proximity of 5Cr-P110 steel is lower than that of 3Cr-P110 steel, which results in a lower precipitation rate and higher dissolution rate of the film on 5Cr-P110 steel, and a porous, loosely-adherent FeS_1−*x*_ scale formation on the inner layer (Figure 8c). Additionally, the lower pH value in the proximity of Cr-containing steels may also, to some extent, be responsible for the lower content of FeCO_3_ crystals in corrosion products (Figure 3). In low Cr steels, the protection is afforded through the formation of a Cr-rich FeCO_3_ corrosion product (Cr oxi-hydroxide) in sweet production conditions [8,9,10,11,12,13]. On the introduction of H_2_S (sour systems), the formation of FeS_1−*x*_ may interfere with Cr-enriched corrosion scales and make the diffusion fluxes of the species involved in the electrochemical reactions easier, lowering the corrosion performance of Cr-containing steels, especially 5Cr-P110 steel. Therefore, 5Cr-P110 steel presents a higher corrosion rate.

Cr^3+^ + H_2_O → Cr(OH)_3_ + 3H^+^ (2)

[Cr(H_2_O)_6_]^3+^ + H_2_O → [Cr(H_2_O)_5_OH]^2+^ + H_3_O^+^ (3)

## 4. Conclusions 

P110 steel suffered localized corrosion and both 3Cr-P110 and 5Cr-P110 steels exhibited general corrosion in CO_2_-H_2_S environment with high pressure and high temperature. The corrosion rate of 3Cr-P110 (1.08 mm/y) was approximate to that of P110 (1.12 mm/y) and the corrosion rate of 5Cr-P110 (1.57 mm/y) was the highest among the three steels.

The corrosion process of the steels was governed by CO_2_ and H_2_S simultaneously under the test conditions. The outer scales on the three steels mainly consisted of FeS_1−*x*_ crystals. The inner scale on P110 steel was composed of amorphous FeS_1−*x*_ and FeCO_3_. However, the inner scale on both 3Cr-P110 and 5Cr-P110 steels comprised amorphous FeS_1−*x*_, Cr(OH)_3_, and FeCO_3_. The inner layers attached directly to the steel surface were apparently denser than outer layers. The more chromium the steel contains, the more chromium compounds the corrosion products contain. The addition of chromium in steels increases the uniformity of the Cr-enriched corrosion scales, eliminates the localized corrosion, but cannot decrease the general corrosion rates. Under test condition, the formation of FeS_1−*x*_ may interfere with Cr-enriched corrosion scales and reduce their protective properties.

However, it is still unclear how H_2_S interferes with the Cr-enriched corrosion film formation. As a consequence of the complexity of CO_2_/H_2_S corrosion, there is a need to carry out further study in order to clarify the mechanism involved in the chromium effect on the corrosion behavior and to develop novel low-chromium steels of both good CO_2_/H_2_S corrosion resistance and low cost.

## Figures and Tables

**Table 1:** Chemical compositions of the tested steels (wt %).

Steel	C	Si	Cr	Mn	Mo	Ni	P	S	V	Fe
P110	0.25	0.29	0.15	0.76	0.27	0.032	0.009	0.004	0.004	Bal.
3Cr-P110	0.26	0.27	2.99	0.58	0.19	0.043	0.011	0.004	0.008	Bal.
5Cr-P110	0.25	0.23	5.11	0.54	0.21	0.041	0.008	0.007	0.009	Bal.

**Table 2:** Binding energies of Fe 2p, O 1s, S 2p, and Cr 2p for inner scales on P110, 3Cr-P110, and 5Cr-P110 samples exposed to 3.5 wt % NaCl solution with CO_2_ and H_2_S (*P*_CO_2__ = 5 MPa, *P*_H_2_S_ = 0.2 MPa, 90 °C, 1 m/s, 360 h). All binding energies are accurate to within ±0.2 eV or less based on three measurements per sample.

Element	P110	3Cr-P110	5Cr-P110
C 1s	284.8 (adventitious) [28]	284.8 (adventitious) [28]	284.8 (adventitious) [28]
	286.4 (adventitious) [28]	286.5 (adventitious) [28]	286.5 (adventitious) [28]
	289.1 (FeCO_3_) [2,27]	289.0 (FeCO_3_) [2,27]	289.1 (FeCO_3_) [2,27]
O 1s	532.0 FeCO_3_ [2,27,28]	530.2 (Cr_2_O_3_ ) [32]	530.2 (Cr_2_O_3_ ) [32]
	-	531.9 (FeCO_3_) [27]	531.2 Cr(OH)_3_ [9]
	-	532.4 (Cr(OH)_3_) [32]	531.9 (FeCO_3_) [27]
	-	-	532.4 (Cr(OH)_3_) [32]
S 2p	162.2 (FeS_1−*x*_) [29]	162.3 (FeS_1−*x*_) [29]	162.3 (FeS_1−*x*_) [29]
	163.7 (elemental sulfur) [3]	163.9 (elemental sulfur) [3]	163.9 (elemental sulfur) [3]
	168.9 (adventitious) [30]	169.2 (adventitious) [30]	168.9 (adventitious) [30]
Fe 2p	710.4 (FeCO_3_) [9,27]	710.4 (FeCO_3_) [9,27]	707.3 (iron sulfide) [31]
	712.1 (FeS_1−*x*_) [29]	712.1 (FeS_1−*x*_) [29]	710.4 (FeCO_3_) [9,27]
	714.1 (FeCO_3_) [9,27]	714.9 (FeCO_3_) [9,27]	712.1 (FeS_1−*x*_) [29]
	724.9(FeCO_3_) [9,27]	724.6(FeCO_3_) [9,27]	724.9(FeCO_3_) [9,27]
Cr 2p	-	577.4 (Cr(OH)_3_) [9,32,33]	577.4 (Cr(OH)_3_) [9,32,33]
	-	586.9 (Cr(OH)_3_) [9,32,33]	578.1(Cr_2_O_3_) [32]
	-	-	586.9 (Cr(OH)_3_) [9,32,33]
